# Circular RNA HECTD1 knockdown inhibits transforming growth factor-beta/ small mothers against decapentaplegic (TGF-β/Smad) signaling to reduce hypertrophic scar fibrosis

**DOI:** 10.1080/21655979.2022.2048771

**Published:** 2022-03-04

**Authors:** Xiaojing Ge, Yute Sun, Youzhi Tang, Jing Lin, Fang Zhou, Gang Yao, Xin Su

**Affiliations:** Department of Plastic and Burn Surgery, The First Affiliated Hospital of Nanjing Medical University, Nanjing, Jiangsu Province, China

**Keywords:** Hypertrophic scars, circHECTD1, TGF-β, miR-142-3P, HMGB1

## Abstract

Scars are nearly impossible to avoid after a skin injury, but despite advancements in the treatment modalities, they remain a clinical problem, especially hypertrophic scars (HS). Many studies include the mechanism of formation and inhibition of HS, but it is not fully understood yet. Circular RNA HECTD1 (circHECTD1), for the first time, has been found to have roles in HS physiology. We determined the relative circHECTD1 levels in HS fibrous cells and tissues by RT-qPCR. Afterward, the effect of circHECTD1 knockdown on the proliferation, migration, invasion, fibrosis, and Transforming Growth Factor-beta/small mothers against decapentaplegic (TGF-β/Smad) signaling was studied using CCK-8, wound healing, Transwell, and western blot assays. After the role of circHECTD1 was clarified, its targeted micro RNA (miR) was predicted using the Starbase database, and we constructed a miR-142-3p mimic to study the details of its regulation mechanism. We used the TargetScan database to predict the downstream target high mobility group box 1 (HMGB1) of miR-142-3p, and the luciferase report assay verified the binding, and then its effect was determined by RT-qPCR. circHECTD1 is highly expressed in HS tissues and human skin hypertrophic scar fibroblasts (HSF); its loss of function inhibits cell proliferation, migration, invasion, fibrosis, and TGF-β/Smad signaling. However, miR-142-3p inhibitor reverses the effect of circHECTD1 on all the above-mentioned aspects, including HMGB1 expression. In conclusion, circHECTD1 knockdown interrupts TGF-β/Smad signaling through miR-142-3p/HMGB1 and suppresses scar fibrosis.

## Graphical abstract

**Figure:**
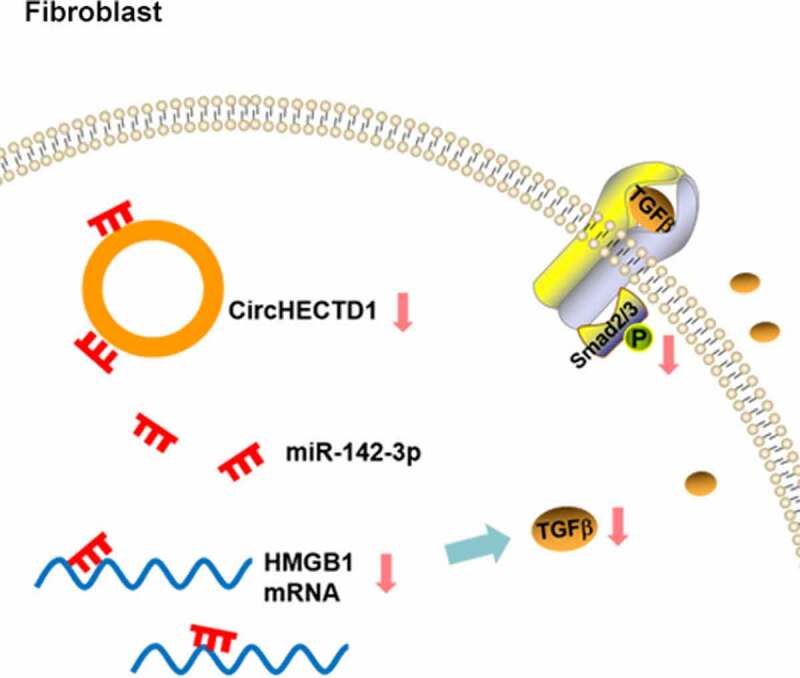

## Introduction

Hypertrophic scar (HS) is a post-traumatic fibrous tissue with excessively growing scars accompanied by dark red/purple coloration, itching, or pain, which raises physical and psychological discomfort in patients [1]. Fibroblasts are cells activated in this process of wound healing, that migrate from the edge of the wound toward the center and transdifferentiate into myofibroblasts. Then, a mass of extracellular matrix (ECM) is synthesized, forming HS [2].

Transforming Growth Factor beta 1 (TGF-β1) is a cytokine involved in HS pathogenesis. TGF-β receptor types I (TGF-βR1) and II (TGF-βR2) are dominant signaling molecules [3]. In wound healing, high TGF-β1 promotes tissue regeneration, and a gradual increase of TGF-β1 activates a variety of intracellular signals, such as small mothers against decapentaplegic (Smad) and mitogen-activated protein kinase (MAPK) pathways. The activation of these pathways sets off a cascade of reactions and stimulates TGF-β1 release, which leads to a continuous autocrine positive feedback loop. This causes the overproduction of matrix proteins, which results in fibrosis [^4–6^]. TGF-β signaling is involved in the formation of HSs [3,7]. Hence, inhibition of TGF-β signaling may affect HS physiology.

Until 2010, circular RNAs (circRNAs) were deemed to be a by-product of splicing [^8–10^]. Due to the progress of RNA high-throughput sequencing, circRNAs were found to be widespread with a variety of biological functions [^11–16^], such as protein translation. Nevertheless, our knowledge of circRNA may remain limited. circHECTD1, a circRNA, is known to promote pulmonary fibrosis [17,18], and its role in scar fibrosis is not completely elucidated. According to previous research, it participates in activating astrocytes by binding with micro RNA (miR)-142-3p [19]. It happens that miR-142-3p has been found to engage in the process of fibrosis and inhibit TGF-β signaling [20,21].

Therefore, circHECTD1 inhibition of TGF-β signaling by binding to miR-142-3p needs to be verified through experiments. Lee et al. found that high mobility group box 1 (HMGB1) promotes skin fibrosis, and can promote TGF-β signaling [22]. To determine how miRNA-142-3p affects TGF-β signaling, we used the TargetScan database to predict the target HMGB1. We evaluated whether miR-142-3p can inhibit the expression of TGF-β by blocking HMGB1 expression. Together, this study aimed to reveal the role and the mechanism of circHECTD1 in scar fibrosis.

## Methods and materials

This study was approved by the Ethics Committee of The First Affiliated Hospital of Nanjing Medical University. Informed, written consent was taken from each participant.

### Tissue samples

HS fibrous skin samples were collected from patients who underwent plastic surgery in our hospital. Meanwhile, the patients’ normal skin samples were obtained during the surgery. The skin tissue samples were treated as previously described [23]. Briefly, the samples were treated with chloramphenicol and then washed with PBS. Then they were digested with 2 mg/ml sterile composite collagenase (Merck KGaA, Germany) at 4°C overnight, filtered through a 200-mesh filter, centrifuged at 1,500 rpm for 15 min. Then, 10% DMEM (Gibco) was added to the precipitate and mixed well, then transferred onto petri dishes for culture.

### Cell culture

When cells reached 90% confluence, they were passaged. The cells were digested with 0.25% trypsin (Thermo Fisher Scientific), the cell suspension was centrifuged at 1,500 rpm for 5 min. Then DMEM containing 10% FBS (Thermo Fisher Scientific) was added to the pellet, and the cells were counted and passaged at 1:4. Human skin fibroblasts (HDF) and human skin HS fibroblasts (HSF) were maintained in an incubator with 5% CO_2_ at 37°C as previously described [24]. The cells used in the experiment were 3–6 generations.

### Real-Time Quantitative Reverse Transcription PCR (RT-qPCR)

RT-qPCR was carried out as previously described [25]. Briefly, total RNA from fibroblasts or skin samples was isolated using TRIzol® reagent (Invitrogen). Complementary DNA was primed using a Sensiscript RT kit (Takara Biotechnology, Osaka, Japan) followed by QuantiTect SYBR Green PCR Kit (Qiagen) for RT-qPCR. The 2^−ΔΔCt^ method [26] was used with normalization using β-actin or U6. Primers are listed in Table 1.

**Table 1.** Sequences of the primers

Gene	Sequence
CircHECTD1	F:5’-ACGGTTGTACGCAAGGTTGA-3’
	R:5’-GGCGCTCTCTCATGATCTCC-3’
Collagen I	F:5’-GTGCTAAAGGTGCCAATGGT-3’
	R:5’-ACCAGGTTCACCGCTGTTAC-3’
Collagen II	F:5’-CACACTCAAGTCCCTCAACAA-3’
	R:5’-AGTAGTCTCCACTCTTCCACTC-3’
a-SMA	F:5’-TGTTCCAGCCATCCTTCATC-3’
	R:5’-GCAATGCCAGGGTACATAGT-3’
HMGB1	F:5’-CAGAACAGAAATACATCTCAGGGC-3’
	R:5’-TCGTGCACCGAAAGTTTCAA-3’
β-actin	F:5’-CGGGAAATCGTGCGTGAC-3’
	R:5’-CAGGAAGGAAGGCTGGAAG-3’
miR-142-3p	F:5’-CTGTGTAGTGTTTCCTACTTTA-3’
	R:5’-GTGCAGGGTCCGAGGT-3’
U6	F:5’-CTCGCTTCGGCAGCACA-3’
	R:5’-AACGCTTCACGAATTTGCGT-3’

### Cell transfection

Fibroblasts were plated into 6-well plates until they reached 70–80% confluence before transfection. Small interfering(si)RNA-CircHECTD1, miR-142-3p mimic, miR-142-3p inhibitor (GenePharma, Shanghai, China), and their controls were transfected into HSF cells with Lipofectamine® 2000 reagent (Thermo Fisher Scientific) as previously indicated [3].

### Cell Counting Kit-8 (CCK-8)

The CCK-8 assay was performed to determine cell proliferation as previously described [27]. Briefly, fibroblasts from different groups were seeded in a 96-well plate and cultured for 24, 48, 72, and 96 h at 37°C, before the CCK-8 solution (AbMole, China) was added. Then the fibroblasts were cultured for another hour followed by the measure of absorbance using a microplate reader (450 nm; Thermo Fisher Scientific).

### Wound healing assay

This assay was performed to determine cell migration as previously described [28]. Briefly, fibroblasts from different groups were placed in 6-well plates and incubated at 37°C to reach ~90% confluence. A tip was used to make a scratch. PBS was used to clear the scratched-off fibroblasts. Fibroblasts were photographed at 0 and 24 h under an inverted microscope (Olympus, Tokyo, Japan). The closure area of the wound was analyzed using Image J 1.52 v software (National Institutes of Health).

### Immunofluorescence assay

This assay was performed as previously described [29]. Briefly, fibroblasts were seeded in 6-well plates and incubated at 37°C to reach ~90% confluence. Then they were subjected to 4% formaldehyde for immobilization. Next, 0.1% Triton X-100 was added and fibroblasts were permeabilized for 15 min. The fibroblasts were blocked with 5% BSA (Merck KGaA, Germany) for 30 min, and α-SMA antibody (Thermo Fisher Scientific) overnight at 4°C, followed by FITC-labeled Goat anti-rabbit secondary antibody (Thermo Fisher Scientific) for 1 h. Counterstaining with DAPI was continued for 5 min for the nuclei, and images were obtained under a fluorescence microscope (Nikon, Japan).

### Transwell assay

This assay was performed to determine cell invasion as previously described [30]. Briefly, fibroblasts in serum-free DMEM were seeded into the upper chamber which was pre-coated with Matrigel (Merck KGaA, Germany), while a medium containing 10% FBS was loaded into the lower chamber. Fibroblasts on the lower side, that crossed the membrane, were first treated with 4% formaldehyde after 24 h-incubation. Next, 0.1% crystal violet solution was applied for dyeing. Five arbitrary fields were selected, and the number of fibroblasts was counted under an inverted microscope.

### Western blotting

Total protein was extracted from the cultured fibroblasts and homogenized in RIPA lysis buffer (Solarbio, Beijing, China). Total protein was quantified using the BCA method (Beyotime, Shanghai, China). Protein samples were then separated through SDS-PAGE and shifted onto PVDF membranes (Solarbio, Beijing, China). These membranes were first blocked with 5% nonfat milk, then washed with TBST. These blots were kept into respective antibodies (1:1,000 diluted (anti-collagen I, anti-collagen II, anti-α-SMA, anti-TGF-β, anti-p-SMAD2, anti-p-SMAD3, anti-SMAD2, anti-SMAD3); Thermo Fisher Scientific) at 4°C overnight. They were cut in strips, which were incubated with an HRP-conjugated anti-rabbit antibody (1:20,000 diluted; Thermo Fisher Scientific) at room temperature for 1 h. To visualize proteins, the BeyoECL Plus kit (Beyotime, Shanghai, China) was used. Image J 1.52 v software was applied to measure the gray values as previously indicated [31].

### Nuclear and cytoplasmic separation

The nuclear fraction of fibroblasts was extracted as previously indicated [32] using a PARIS^TM^ kit (Ambion, USA). Cultured fibroblasts were washed once in PBS and subjected to lysis buffer in an ice bath for 15 min. The sample was centrifuged and the supernatant was collected. Otherwise, the pellet was lysed in nuclei lysis buffer (Saint, Shanghai, China) in an ice bath for 20 min. The extract was collected after centrifugation. U6 and GAPDH were used as the controls.

### Luciferase reporter assay

Fibroblasts were seeded in the 24-well plates. CircHECTD1 wild-type and mutant-type luciferase vectors were co-transfected with miR-142-3p mimic using Lipofectamine® 2000 reagent. Similarly, HMGB1 vectors were constructed. The Luciferase Reporter Gene Assay kit (Beyotime, Shanghai, China) was used in accordance with the instruction manual. The relative luciferase activity was measured after 48 h and normalized to *Renilla* luciferase activity as previously described [33].

### Database and statistical analysis

The Starbase database [34] was used to predict the binding of circHECTD1 to miR-142-3p. The miR-142-3p target, HMGB1, was predicted based on the TargetScan database [35]. Experimental data were analyzed with GraphPad 7.0 software and displayed as mean ± SD of three replicates. Student’s *t*-test (two groups), one-way ANOVA followed by Tukey’s *post-hoc* test (multiple groups) were used to evaluate differences. Results were considered of statistical significance if p < 0.05.

## Results

The present study aimed to reveal the role and the mechanism of circHECTD1 in scar fibrosis. circHECTD1 was found to be highly expressed in HS tissues and HSF; its loss of function inhibited cell proliferation, migration, invasion, fibrosis, and TGF-β/Smad signaling. Moreover, miR-142-3p inhibitor reversed the effect of circHECTD1 on all the above-mentioned aspects, including HMGB1 expression. In conclusion, circHECTD1 knockdown interrupted TGF-β/Smad signaling through miR-142-3p/HMGB1 and suppressed scar fibrosis.

### The expression of circHECTD1 in cells and human tissues

**Figure 1.**
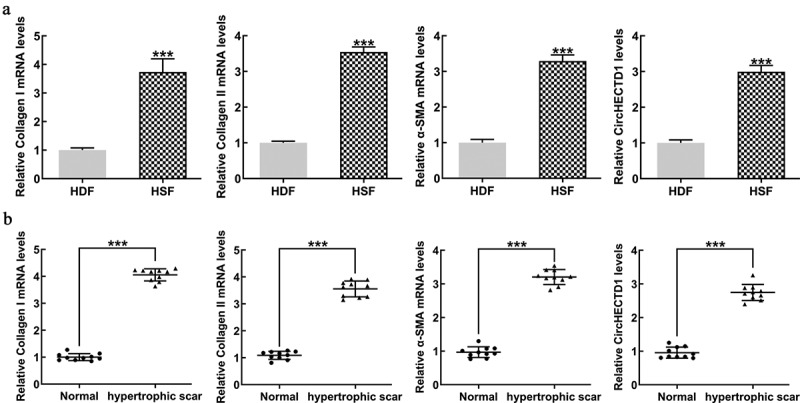
circHECTD1 expression in cells and human tissues. (a) Expression of collagen I, collagen II, α-SMA mRNAs, and circHECTD1 in HDF and HSF detected by RT-qPCR. ***P < 0.001 versus HDF; n = 3. (b) Expression of collagen I, collagen II, α-SMA mRNA, and circHECTD1 in normal skin tissues and HS fibrous skin tissues was detected by RT-qPCR. ***P < 0.001 versus normal; n = 10.

We evaluated the mRNA expression of collagen I, collagen II, and α-SMA in all tissue samples by RT-qPCR. The levels of all three proteins in HSF were markedly higher than those in HDF. circHECTD1 was also higher in HSF (Figure 1(a)). Protein expression in HS fibrous skin tissue samples was compared with healthy skin samples. The result shows that these proteins in scar fibrous skin tissue were significantly higher than in normal skin (Figure 1(b)).

### circHECTD1 knockdown inhibits proliferation, migration, invasion, and fibrosis

**Figure 2.**
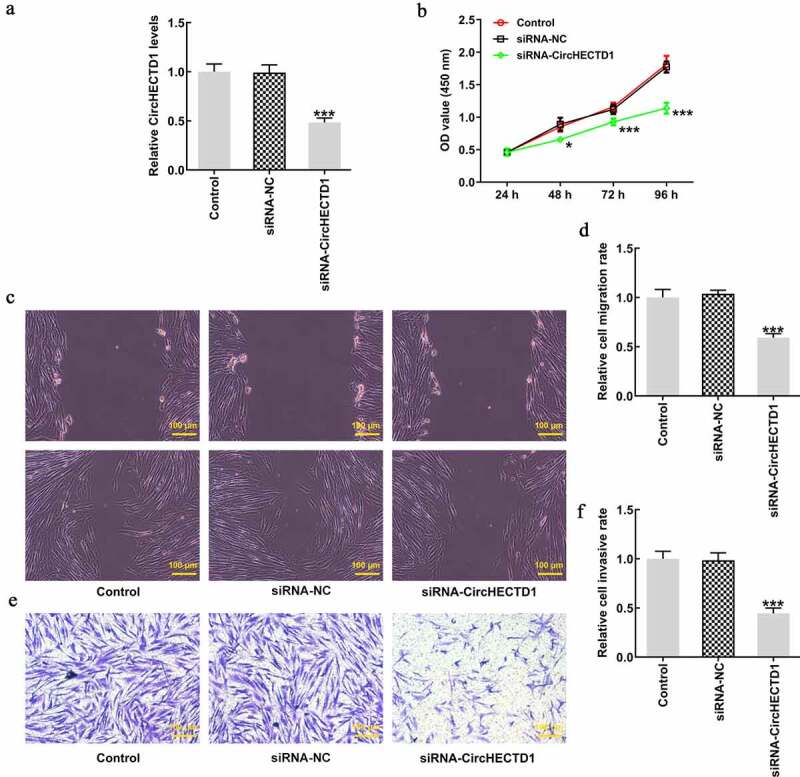
circHECTD1 knockdown inhibits the proliferation, migration and invasion of HSF. (a) Knockdown in HSF detected by RT-qPCR. ***P < 0.001 versus siRNA-NC; n = 3. (b) Proliferation expressed as OD value using CCK8 assay. *P < 0.05, ***P < 0.001 versus 24 h; n = 3. (c) Cell migration and (d) invasion detected by wound healing and Transwell assay, respectively. ***P < 0.001 versus siRNA-NC; n = 3.

**Figure 3.**
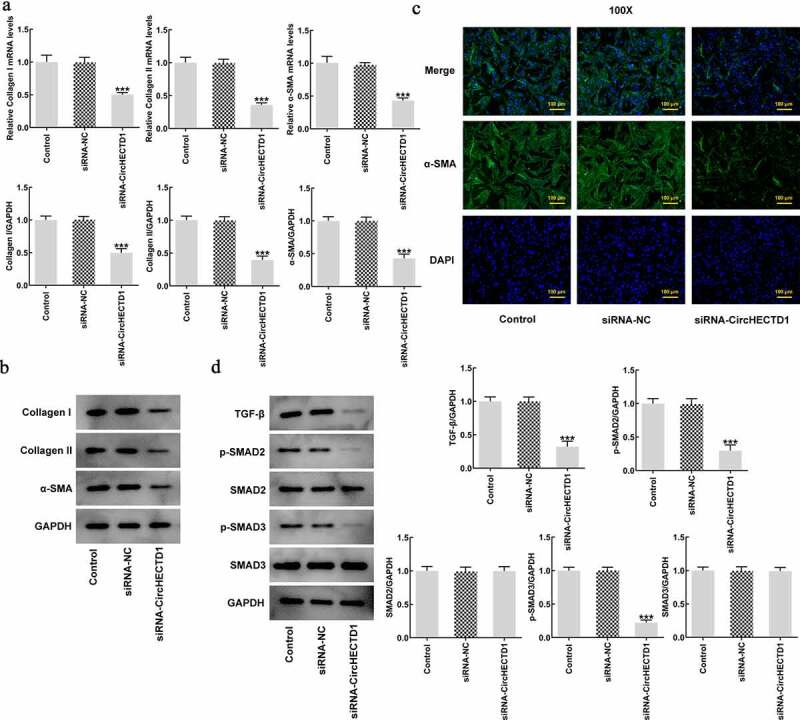
circHECTD1 knockdown inhibits the fibrosis and the expression of TGF-β/SMAD. (a) Collagen I, collagen II, and α-SMA mRNA expression in the three groups detected by RT-qPCR, and (b) western blot. (c) α-SMA expression detected by immunofluorescence. (d) TGF-β, p-SMAD2, p-SMAD3, SMAD2, SMAD3 expression detected by western blot. ***P < 0.001 versus siRNA-NC; n = 3.

RT-qPCR was applied to assess the degree of circHECTD1 knockdown in HSF. Compared with siRNA-NC, the expression of circHECTD1 in siRNA-circHECTD1 transfected cells decreased (Figure 2(a)). Therefore, these cells transfected with siRNA-circHECTD1 were employed in the following experiments. The proliferation of control, siRNA-NC, and siRNA-circHECTD1 was examined using a CCK8 assay. The values at 24, 48, 72, 96 h in all groups are displayed in a line chart. The result indicated that circHECTD1 knockdown inhibited the proliferation of HSF (Figure 2(b)). Afterward, the migration ability of the three groups was assessed with wound healing, along with Transwell assay for invasion ability, which both decreased in the siRNA-circHECTD1 group (Figure 2(c,d)). Moreover, RT-qPCR and western blotting were used to determine collagen I, collagen II, and α-SMA mRNA and protein expression. In the three groups, they all showed a decrease in the siRNA-circHECTD1 group (Figure 3(a,b)). Meanwhile, immunofluorescence was also used to evaluate the expression of α-SMA. The fluorescence of the siRNA-circHECTD1 group was obviously weaker than the negative control group (Figure 3(c)). Since α-SMA is a differentiation marker, the result indicates that knockdown with circHECTD1 inhibits HSF fibrosis.

### circHECTD1 knockdown inhibits the expression of TGF-β/SMAD

TGF-β signaling pathway involves phosphorylation of SMADs. Roberts et al. found that TGF-β plays an important role in mediating wound healing and scar formation through SMAD3 [36]. Therefore, to investigate the role of circHECTD1 in TGF-β/SMAD signaling, TGF-β, p-Smad2, p-Smad3, Smad2, Smad3 were determined using western blotting. circHECTD1 knockdown decreased the expressions of TGF-β, p-Smad2, p-Smad3, while Smad2 and Smad3 remained unvaried (Figure 3(d)). This indicates that circHECTD1 knockdown could inhibit the expression of TGF-β/SMAD signaling in HSF.

### circHECTD1 knockdown promotes miR-142-3p expression

**Figure 4.**
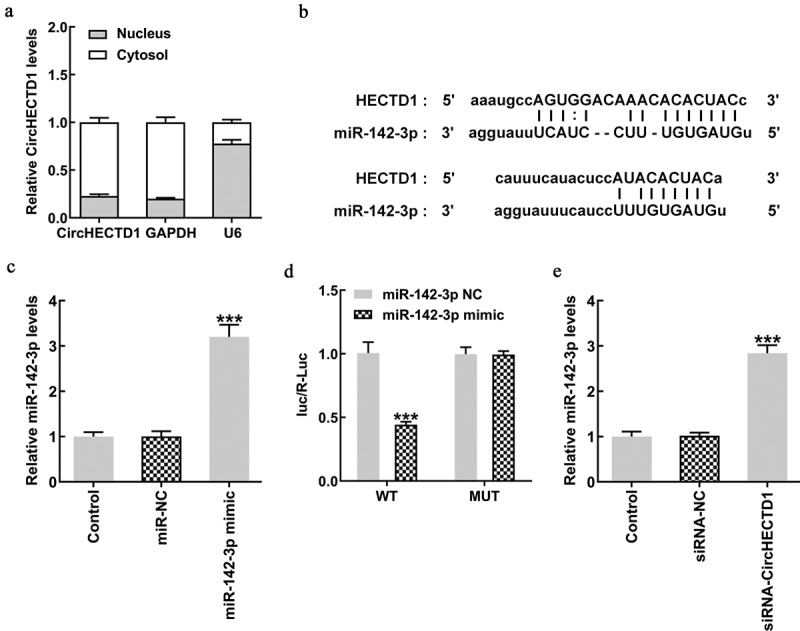
circHECTD1 interferes with miR-142-3p, promoting its expression. (a) circHECTD1 expression in the cytoplasm and nucleus. (b) circHECTD1-miR-142-3p binding predicted by Starbase database. (c) miR-142-3p expression detected by RT-qPCR. ***P < 0.001 versus miR-NC; n = 3. (d) circHECTD1-miR-142-3p binding detected by luciferase reporter assay. ***P < 0.001 versus wt-circHECTD1 + miR-142-3p NC; n = 3. (e) miR-142-3p expression detected by RT-qPCR in the three groups. ***P < 0.001 versus siRNA-NC; n = 3.

We found circHECTD1 expression to be higher in the cytoplasm (Figure 4(a)). The Starbase database was used to predict the targeted binding of circHECTD1 to miR-142-3p (Figure 4(b)). First, miR-142-3p expression was evaluated using RT-qPCR. The expression of the mimic group was found to be higher in contrast with the NC group (Figure 4(c)). In addition, luciferase activity of circHECTD1 wild-type in the miR-142-3p mimic group was decreased in comparison with that in the miR-142-3p NC group, and the mutant-type had no significant change (Figure 4(d)). This shows that circHECTD1 targets miR-142-3p, and the expression levels of miR-142-3p in these three groups were determined. As expected, miR-142-3p expression in the siRNA-circHECTD1 group was markedly higher (Figure 4(e)).

### miR-142-3p inhibitor reverses the effect of circHECTD1 knockdown on proliferation, invasion, and migration

**Figure 5.**
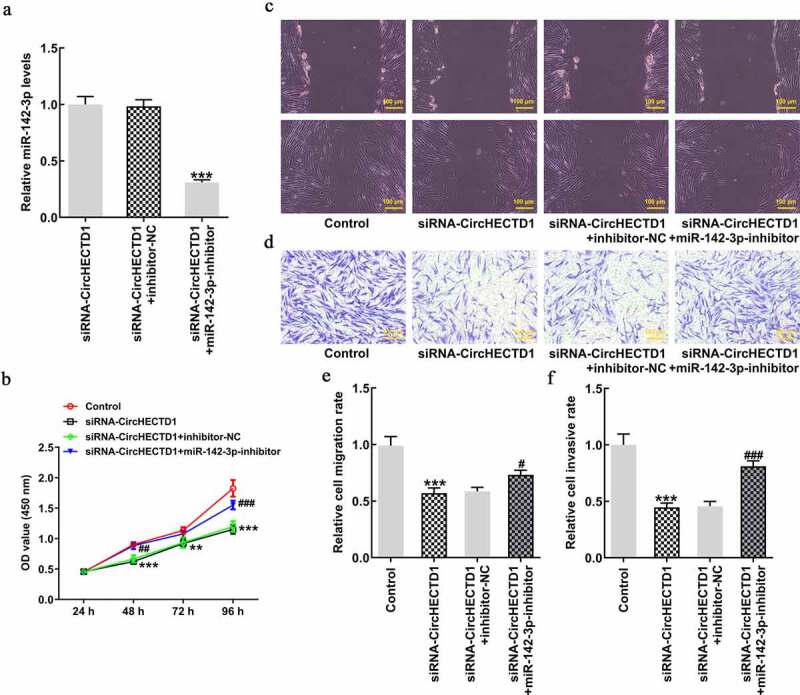
miR-142-3p inhibitor reverses the effect of circHECTD1 knockdown on proliferation, invasion, and migration. (a) The miR-142-3p inhibitor was constructed and its effectiveness checked by RT-qPCR. ***P < 0.001 versus siRNA-circHECTD1 + inhibitor-NC; n = 3. (b) Proliferation expressed as OD values using CCK8 assay. **P < 0.01, ***P < 0.001 versus Control; ^##^P < 0.01, ^###^P < 0.001 versus siRNA-circHECTD1 + inhibitor-NC; n = 3. (c) Cell migration and (d) invasion detected by wound healing and Transwell assay, respectively. ***P < 0.001 versus Control; ^#^P < 0.05, ^###^P < 0.001 versus siRNA-circHECTD1 + inhibitor-NC; n = 3.

HSFs were transfected with miR-142-3p inhibitor, and RT-qPCR result revealed that the expression of miR-142-3p in siRNA-circHECTD1 group with miR-142-3p inhibitor decreased (Figure 5(a)). Subsequently, the result of the CCK8 assay indicated that miR-142-3p inhibitor reversed the inhibitory effect of siRNA-circHECTD1 on proliferation (Figure 5(b)). Moreover, wound healing and Transwell assays showed that the addition of miR-142-3p inhibitor reversed the inhibition of migration and invasion, respectively, caused by siRNA-circHECTD1 (Figure 5(c–f)).

### miR-142-3p inhibitor reverses the effect of circHECTD1 knockdown on fibrosis and expression of TGF-β/SMAD signaling

**Figure 6.**
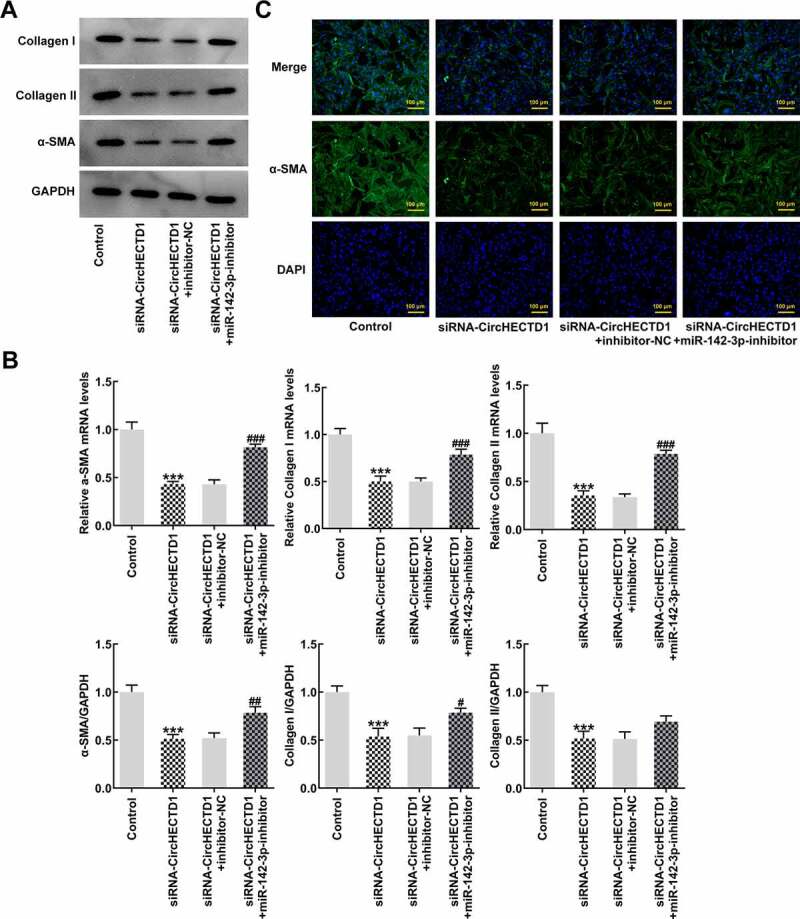
miR-142-3p inhibitor reverses the effect of circHECTD1 knockdown on fibrosis and TGF-β/SMAD signaling. (a) Collagen I, collagen II, and α-SMA mRNA expression detected by western blot, and (b) RT-qPCR. (c) α-SMA expression detected by immunofluorescence. ***P < 0.001 versus Control; ^#^P < 0.05, ^##^P < 0.01, ^###^P < 0.001 versus siRNA-circHECTD1 + inhibitor-NC; n = 3.

**Figure 7.**
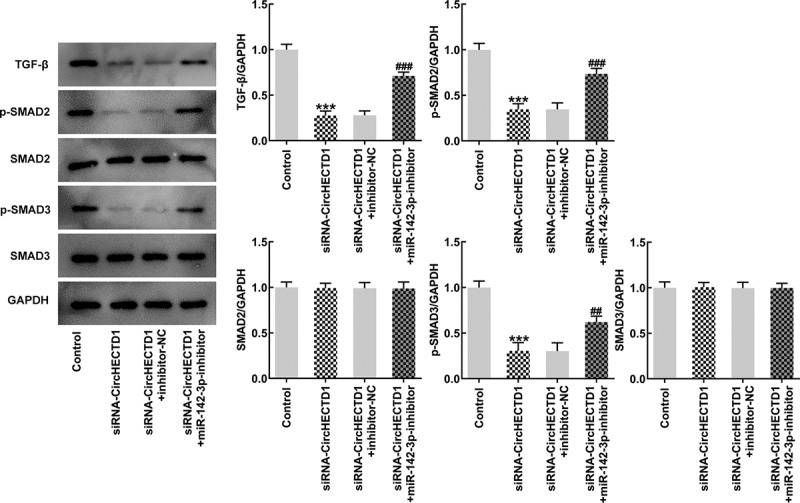
miR-142-3p inhibitor reverses the effect of circHECTD1 knockdown on fibrosis and TGF-β/SMAD signaling. TGF-β, SMADs expression detected by western blot. ***P < 0.001 versus control, ^##^P < 0.01, ^###^P < 0.001 versus siRNA-circHECTD1 + inhibitor-NC; n = 3.

To verify whether the miR-142-3p inhibitor also reverses fibrosis and TGF-β/SMAD signaling, RT-qPCR and western blotting were employed to determine the expression of collagen I, collagen II, and α-SMA, accompanied by immunofluorescence for α-SMA. The results demonstrated that the expression in the siRNA-circHECTD1 with NC group decreased, and the trend reversed when miR-142-3p was inhibited (Figure 6(a,b)). And the fluorescence of the miR-142-3p inhibitor group was greater than the NC group (Figure 6(c)). In addition, the expression levels of TGF-β and SMADs were assessed using western blotting. TGF-β, p-Smad2, and p-Smad3 levels in the miR-142-3p inhibitor group were elevated, which again activated *TGF-β/SMAD* signaling (Figure 7).

### circHECTD1 acts on scar fibrosis through miR-142-3p/HMGB1

**Figure 8.**
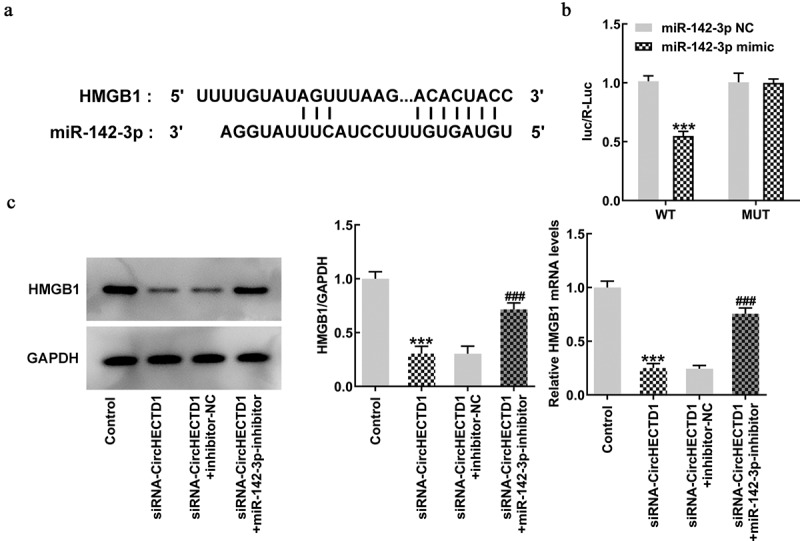
circHECTD1 acts on scar fibrosis through miR-142-3p/HMGB1. (a) TargetScan database shows complementarity between miR-142-3p and HMGB1 3’-UTR. (b) miR-142-3p targets HMGB1 verified by luciferase reporter assay. ***P < 0.001 versus wt-HMGB1 + miR-142-3p NC; n = 3. (c) HMGB1 expression detected by RT-qPCR and western blot. ***P < 0.001 versus control, ^###^P < 0.001 versus siRNA-circHECTD1 + inhibitor-NC; n = 3.

To verify the direct targets of miR-142-3p, TargetScan was run to predict the putative target of miR-142-3p. It was found that there was complementarity between miR-142-3p and HMGB1 3’-UTR (Figure 8(a)). Therefore, a luciferase reporter assay was conducted to affirm if miR-142-3p targets HMGB1. Luciferase activity in only the wt-HMGB1 with miR-142-3p mimic group decreased, indicating possible binding between miR-142-3p and HMGB1 (Figure 8(b)). Finally, RT-qPCR and western blotting were used to detect the expression of HMGB1 to verify whether miR-142-3p inhibitor reverses the effect of circHECTD1 knockdown on HMGB1 expression. Just as anticipated, HMGB1 expression decreased in the siRNA-circHECTD1 group and the reduction was reversed when miR-142-3p was inhibited (Figure 8(c)).

## Discussion

HS is a fibrotic disease characterized by the activation and over-proliferation of fibroblasts and is often considered a benign skin tumor [37]. It can cause continuous itching and pain. In severe cases, sequelae, such as organ dysfunction or physical deformity may occur, which arises physical and psychological burdens [38,39]. Despite treatments, such as laser, resection, and compression [40,41], it is difficult to completely cure [42]. Establishing the mechanism of HS formation requires further investigation. Since the excessive abnormal proliferation of fibroblasts majorly contributes to the occurrence and development of HS [^43–45^], inhibiting the proliferation, migration, and invasion of HSF could be an effective way to treat HS.

Collagen, an ECM component produced by fibroblasts, interacts with integrin receptors to regulate gene expression, cell proliferation, and even differentiation. RT-qPCR results showed that collagen I, collagen II, and α-SMA mRNAs were high in HSF. Moreover, human HS fibrous skin tissue also showed high expression of these mRNAs.

CircRNAs are widely expressed non-coding RNAs, playing vital roles in glioma, liver cancer, gastric cancer, and lung fibrosis. CircHECTD1 can promote pulmonary fibrosis in silicosis by regulating the activation and migration of fibroblasts [17]. In healthy human skin tissue and HS, using high-throughput RNA sequencing, 11 circRNA genes, showing significant differential expression, were filtered from 3649 lncRNA genes [46]. We found that circHECTD1 was up-regulated in HSF. Thus, using RT-qPCR, western blot, and immunofluorescence, we found that loss of function of circHECTD1 could inhibit the proliferation, invasion, and migration of HSF, thus inhibiting fibrosis.

TGF-β can directly provoke the generation of proteins, such as collagen and fibronectin, and adjust their stability by changing the balance between matrix metalloproteinases (MMPs) and their inhibitors. TGF-β1 was first thought to induce the proliferation of normal mouse fibroblasts and accelerate the closure of incision wounds in mice [47]. It activates Smad family proteins by binding to TGF-β1 R. The activated Smad proteins transduce the TGF-β1 signal to the nucleus and regulate gene expression [48]. A previous study indicated that the collagen expression in Smad3 knockout rats was declined compared with that in the normal rat, and fibrosis was slowed down [49]. To find out if circHECTD1 affects TGF-β/Smad signaling, the expression of TGF-β and Smads in transfected HSF was assessed. The decline in expression indicated that circHECTD1 knockdown inhibited TGF-β/Smad signaling. We concluded that CircHECTD1 could affect HSF fibrosis and inhibit TGF-β/Smad signaling, however, the specific mechanism is not known.

CircRNAs sponge miRNAs by competing for miRNA binding sites, thereby reducing the expression of targeted mRNAs. Previous research has shown that miR-142-3p affects lung fibroblasts [50,51]. Exosomes derived from macrophages counteract lung fibrosis by delivering anti-fibrotic miR-142-3-p to alveolar epithelial cells and fibroblasts [52]. Herein, following the confirmation of the association between circHECTD1 and miR-142-3p, the expression of miR-142-3p following circHECTD1 knockdown was evaluated. We found that the knockdown promoted the expression of miR-142-3p, and miR-142-3p inhibitor reversed the effects of circHECTD1 knockdown on proliferation, migration, invasion, fibrosis, and signaling. Our results identified with the results of the aforementioned investigations that have shown miR-142-3p expression inhibits pulmonary fibrosis.

Furthermore, the TargetScan database predicted the miR-142-3p target, HMGB1. The prediction was certified through a luciferase reporter assay. RT-qPCR and western blotting were used to detect HMGB1 expression when circHECTD1 knockdown alone or with miR-142-3p suppression. Apparently, miR-142-3p inhibitor reversed the effect of circHECTD1 on HMGB1 expression. Of note, whether miR-142-3p is the only target of circHECTD1 in scar fibrosis merits further research.

## Conclusion

To sum up, loss of circHECTD1 function inhibits TGF-β/Smad signaling through miR-142-3p/HMGB1 and suppresses a fibrotic phenotype within HS fibroblasts. This article is the first to study the role of circHECTD1 in HS, which enriches the knowledge of HS pathology.

## Data Availability

The data that support the findings of this study are available with the corresponding author upon reasonable request.
